# Advances in genomics and genome editing for improving strawberry (*Fragaria ×ananassa*)

**DOI:** 10.3389/fgene.2024.1382445

**Published:** 2024-04-19

**Authors:** Kaitlyn Vondracek, Fredy Altpeter, Tie Liu, Seonghee Lee

**Affiliations:** ^1^ Gulf Coast Research and Education Center, Institute of Food and Agricultural Sciences, University of Florida, Wimauma, FL, United States; ^2^ University of Florida, Horticultural Sciences Department, Institute of Food and Agricultural Sciences, Gainesville, FL, United States; ^3^ University of Florida, Agronomy Department, Institute of Food and Agricultural Sciences, Gainesville, FL, United States

**Keywords:** CRISPR/Cas genome editing, octoploid strawberry, genome sequencing, transformation, polyploid

## Abstract

The cultivated strawberry, *Fragaria ×ananassa*, is a recently domesticated fruit species of economic interest worldwide. As such, there is significant interest in continuous varietal improvement. Genomics-assisted improvement, including the use of DNA markers and genomic selection have facilitated significant improvements of numerous key traits during strawberry breeding. CRISPR/Cas-mediated genome editing allows targeted mutations and precision nucleotide substitutions in the target genome, revolutionizing functional genomics and crop improvement. Genome editing is beginning to gain traction in the more challenging polyploid crops, including allo-octoploid strawberry. The release of high-quality reference genomes and comprehensive subgenome-specific genotyping and gene expression profiling data in octoploid strawberry will lead to a surge in trait discovery and modification by using CRISPR/Cas. Genome editing has already been successfully applied for modification of several strawberry genes, including anthocyanin content, fruit firmness and tolerance to post-harvest disease. However, reports on many other important breeding characteristics associated with fruit quality and production are still lacking, indicating a need for streamlined genome editing approaches and tools in *Fragaria ×ananassa*. In this review, we present an overview of the latest advancements in knowledge and breeding efforts involving CRISPR/Cas genome editing for the enhancement of strawberry varieties. Furthermore, we explore potential applications of this technology for improving other Rosaceous plant species.

## 1 Introduction

The cultivated strawberry (*Fragaria ×ananassa*) is among the most economically important fruit crops in the world. According to the Food and Agricultural Organization of the United Nations, during 2021, more than nine million tonnes of strawberries were produced from 389,665 ha around the world (FAO, 2023). The cultivated strawberry as it is today resulted from a series of interspecific hybridizations, with the final cross between octoploid progenitors occurring only approximately 300 years ago (Edger et al., 2019; Whitaker et al., 2020). Evolutionary analysis of *Fragaria ×ananassa* suggested genomic contributions of four diploid progenitor species: *Fragaria vesca, Fragaria iinumae, Fragaria viridis*, and *Fragaria nipponica* (Edger et al., 2019; 2020). However, analysis from other groups has suggested that both *F. viridis* and *F. nipponica* are not among the diploid progenitors (Liston et al., 2020; Jin et al., 2023; Lyu et al., 2023; Session and Rokhsar, 2023). As identification of the progenitor species may enable greater prediction of polyploid responses to environmental stress and climate change (Liston et al., 2020), further analysis of the evolutionary history of strawberry will be necessary to confirm the identities of the diploid progenitor species.

*Fragaria ×ananassa* is a highly heterozygous, allo-octoploid species (2n = 8x = 56) with a phased genome length of approximately 780 Mb (Hardigan et al., 2021a; Han et al., 2022). Due to the complex nature of the allo-octoploid genome and high genomic heterozygosity, trait discovery and gene functionalization studies in strawberry are commonly performed in the diploid woodland strawberry, *Fragaria vesca*. *Fragaria vesca* is used as a model system for strawberry research for several reasons: it has a small genome size of approximately 240Mb, has a short life cycle, is relatively responsive to transformation, and is easy to propagate using both seeds and runner cuttings (Oosumi et al., 2006). *Fragaria vesca* is additionally the closest relative of the transcriptionally dominant diploid subgenome of *Fragaria ×ananassa* (Hardigan et al., 2021b), further lending to its value as a model system for strawberry research. While trait discovery and gene functional studies performed in *Fragaria vesca* are useful for inferring mechanisms of commercially valuable traits in the cultivated strawberry, there are also drawbacks to relying on the diploid system. The largest drawback to working with the diploid strawberry is that *Fragaria vesca* tends to be more homozygous at a given locus as compared to *Fragaria ×ananassa*. Martín-Pizarro et al. (2019) identified one such instance of this when they identified at least five alleles of the *FaTM6* locus across the four homoeologous chromosomes of octoploid strawberry and only a single homozygous *FvTM6* locus in the diploid strawberry. Due to the differences in heterozygosity, it can be difficult to determine which allele(s) in *Fragaria ×ananassa* contribute to a phenotype using data from *Fragaria vesca* alone, hindering attempts at genomics-assisted improvement of commercially relevant varieties.

Recent advancements in genome sequencing technology and gene annotation have enabled the assembly of numerous strawberry genomes encompassing a range of ploidy levels (Qiao et al., 2021; Han et al., 2022; Song et al., 2023a). Enabled by these genome assemblies, CRISPR/Cas genome editing is increasingly applied for the study and improvement of key strawberry traits. While not currently widespread due to several challenges, genome editing in allo-octoploid strawberry offers a unique opportunity to precisely modify traits of interest. Alternatively, tools such as RNAi, stable or transient overexpression of transgenes have been utilized to validate gene function and support identification of candidate genes. Plant breeding has also made use of genomics-assisted techniques such as marker-assisted selection and genomic selection for varietal improvement, enabled by new methods for QTL discovery and analysis.

This review covers the genomic tools and technologies available for genomics-assisted improvement of cultivated strawberries, in addition to multi-omics technologies and their applications in identifying candidate QTLs and genes. Reports of successful CRISPR/Cas genome editing for traits associated with strawberry fruit quality and production are discussed herein. In addition to these reports, this review suggests additional candidates for genome editing based on analyses in strawberry and other crops.

## 2 Advanced genomic resources in strawberry

Genomic complexity of the polyploid genome is one of the major challenges of genome editing in octoploid strawberry. The first high quality, chromosome-scale reference genome of *Fragaria ×ananassa* cv. ‘Camarosa’ was developed in 2019 ​(Edger et al., 2019)​. Before the release of the first octoploid strawberry genome, there were few genome assemblies available for polyploid species, as the homoeologous nature of the subgenomes made assembly of sequences difficult (Wang et al., 2023b). As such, molecular genetic analysis of *Fragaria ×ananassa* prior to 2019 relied on early genome assemblies of *Fragaria vesca*, the first of which was published in 2011 (Shulaev et al., 2011). Recent rapid advancements in genome sequencing technology have resulted in the release of additional annotated octoploid genome sequences of increasing quality (Table 1) (Wang et al., 2023b). Advancements in genome sequencing in strawberry have not been limited only to the octoploid, as new high-quality genomes have also recently been released for the diploid model species, *Fragaria vesca* (Alger et al., 2021; Joldersma et al., 2022). As improvements to genome sequencing technology continue, it will be possible to better understand genomic complexity in not only octoploid strawberry, but in other polyploids as well.

**TABLE 1:** Compilation of publicly available genome assemblies for various *Fragaria* species. (−) indicates value not reported.

Species	Ploidy	Genotype	Genome Name	Sequencing Platform(s)	Assembled genome size (Mb)	Reference
*Fragaria nilgerrensis*	2n = 2x = 14	(−)	*Fragaria nilgerrensis* Genome v1.0	Illumin HiSeq X Ten; PacBio; Hi-C	270.30	Zhang et al. (2020)
	2n = 2x = 14	(−)	*Fragaria nilgerrensis* SCBG Genome v1.0	PacBio	271.90	Feng et al. (2021a)
	2n = 2x = 14	(−)	*Fragaria nilgerrensis* YNU Genome v1.0	PacBio	288.43	Qiao et al. (2021)
*Fragaria pentaphylla*	2n = 2x = 14	(−)	*Fragaria pentapylla* YNU Genome v1.0	PacBio	279.04	Qiao et al. (2021)
*Fragaria mandschurica*	2n = 2x = 14	(−)	*Fragaria mandschurica* YNU Genome v1.0	PacBio	239.83	Qiao et al. (2021)
*Fragaria daltoniana*	2n = 2x = 14	(−)	*Fragaria daltonia* YNU Genome v1.0	Oxford Nanopore PromethION	288.97	Qiao et al. (2021)
*Fragaria viridis*	2n = 2x = 14	(−)	*Fragaria viridis* SCBG Genome v1.0	PacBio	214.60	Feng et al. (2021a)
	2n = 2x = 14	(−)	*Fragaria viridis* YNU Genome v1.0	Oxford Nanopore PromethION	223.08	Qiao et al. (2021)
*Fragaria iinumae*	2n = 2x = 14	(−)	*Fragaria iinumae Genome v1.0 (FII_r1.1)*	Roche 454 GS FLX+	199.63	Hirakawa et al. (2014)
	2n = 2x = 14	(−)	*Fragaria iiunumae* Genome v1.0	PacBio	240.58	Edger et al. (2020)
*Fragaria nubicola*	2n = 2x = 14	(−)	*Fragaria nubicola* SCBG Genome v1.0	PacBio	247.20	Feng et al. (2021a)
*Fragaria orientalis*	2n = 2x = 14	(−)	*Fragaria orientalis* Genome v1.0 (FOR_r1.1)	Roche 454 GS FLX+	214.18	Hirakawa et al. (2014)
*Fragaria bucharica*	2n = 2x = 14	(−)	*Fragaria nubicola* Genome v1.0 (FNU_r1.1)	Roche 454 GS FLX+	203.69	Hirakawa et al., 2014, Tennessen et al., 2014
*Fragaria nipponica*	2n = 2x = 14	(−)	Fragaria nipponica Genome v1.0 (FNI_r1.1)	Roche 454 GS FLX+	206.41	Hirakawa et al. (2014)
	2n = 2x = 14	(−)	*Fragaria nipponica* KIB CAS Genome v1.0	Nanopore GridION 5x	290.90	Jin et al. (2023)
*Fragaria vesca*	2n = 2x = 14	Hawaii-4	*Fragaria vesca* Genome v1.0	Roche 454; Illumina SOLid	209.80	Shulaev et al. (2011)
	2n = 2x = 14	Hawaii-4	*Fragaria vesca* Genome v1.1	Roche 454; Illumina SOLid	209.80	Shulaev et al. (2011)
	2n = 2x = 14	(−)	*Fragaria vesca* Genome v2.0.a1 (Fvb)	Illumina HiSeq 2000	211.70	Tennessen et al. (2014)
	2n = 2x = 14	YW5AF7	*Fragaria vesca* Genome v1.1.a2	Illumina HiSeq 2000	(−)	Darwish et al. (2015)
	2n = 2x = 14	(−)	*Fragaria vesca* Genome 2.0.a2	PacBio	(−)	Li et al. (2018b)
	2n = 2x = 14	Hawaii-4	*Fragaria vesca* Genome v4.0.a1	PacBio; Illumina	220.50	Edger et al. (2018)
	2n = 2x = 14	(−)	*Fragaria vesca* Genome v4.0.a2 (FvH4_v4.0)	PacBio; Illumina	219.00	Li et al. (2019b)
	2n = 2x = 14	CFRA 2339	*Fragaria vesca* (CFRA 2339) Genome v1.0	Oxford Nanopore GridION x 5	229.50	Alger et al. (2021)
	2n = 2x = 14	Yellow Wonder/5AF7	*Fragaria vesca* Yellow Wonder Genome v1.0 (FvYW_v1.0)	Oxford Nanopore PromethION	220.00	Joldersma et al. (2022)
	2n = 2x = 14	Hawaii-4	*Fragaria vesca* ‘Hawaii 4′NAU Genome v1.0	PacBio HiFi	220.80	Zhou et al. (2023)
*Fragaria chiloensis*	2n = 8x = 56	(−)	*Fragaria chiloensis* KIB CAS Genome v1.0	PacBio CCS	839.90 (hap 1); 824.20 (hap 2)	Jin et al. (2023)
*Fragaria virginiana*	2n = 8x = 56	(−)	*Fragaria virginiana* KIB CAS Genome v1.0	PacBio CCS	787.80 (hap 1); 769.20 (hap 2)	Jin et al. (2023)
*Fragaria x ananassa*	2n = 8x = 56	Reikou	*Fragaria x ananassa* Genome v1.0 (FAN_r1.1)	Roche 454 GS FLX+; Illumina GAIIx/HiSeq 1000	697.77	Shirasawa et al. (2021)
	2n = 8x = 56	(−)	*Fragaria x ananassa* Reference Genome v1.0 (FANhybrid_r1.2)	Roche 454 GS FLX+; Illumina GAIIx/HiSeq 1000	173.23	Hirakawa et al. (2014)
	2n = 8x = 56	Camarosa	*Fragaria x ananassa* Camarosa Genome Assembly v1.0.a1	PacBio RSII; Illumina HiSeq X/HiSeq 2500/HiSeq 4000	805.49	Edger et al. (2019)
	2n = 8x = 56	Wongyo 3,115	*Fragaria x ananassa* Cultivar: Wongyo 3,115 (NCBI Accession PRJNA662854)	PacBio Sequel; Illumina NovaSeq 6000	805.7	Lee et al. (2021)
	2n = 8x = 56	Camarosa	*Fragaria x ananassa* Camarosa Genome v1.0.a2 (Reannotation of v1.0.a1)	Publicly available PacBio SMRT and Illumina data	(−)	Liu et al. (2021)
	2n = 8x = 56	Royal Royce	*Fragaria x ananassa* Royal Royce Genome v1.0	PacBio Sequel II; Illumina NovaSeq S4	784.48 (Hap 1); 783.92 (Hap 2); 786.54 (FaRR1)	Hardigan et al. (2021a)
	2n = 8x = 56	FL15.89–25	*Fragaria x ananassa* FL 15.89–25 Genome v1.0	PacBio Sequel II	827.30 (Hap F12); 839.40 (Hap Bea)	Fan et al. (2022)
	2n = 8x = 56	Florida Brilliance	*Fragaria x ananassa* Florida Brilliance Genome v1.0	PacBio HiFi; Hi-C	784.90 (Hap 1); 781.00 (Hap 2)	Han et al. (2022)
	2n = 8x = 56	Yanli	*Fragaria x ananassa* Yanli Genome v1.0	Pacbio HiFi; Illumina Novaseq 6000; Illumina HiSeq X Ten	825.00 (Hap 1); 808.00 (Hap 2)	Mao et al. (2023)
	2n = 8x = 56	Benihoppe	*Fragaria x ananassa* Cultivar: Benihoppe (NCBI Accession PRJNA970713)	PacBio Sequel II; Illumina NovaSeq 6000	852.00 (Hap 1); 821.00 (Hap 2)	Song et al. (2023b)

Recently, multiple single nucleotide polymorphism (SNP) arrays for octoploid strawberry have been developed to assist with quantitative trait loci (QTL) discovery, including 50K and 90K arrays (Bassil et al., 2015; Verma et al., 2017). Using these tools to identify SNPs correlated to QTLs enables the development of molecular markers which can be utilized in breeding (Jung et al., 2017). Marker assisted selection (MAS) is a method of precision genomics-assisted breeding which relies on the implementation of genetic markers and trait associations to inform selection (Collard and Mackill, 2008). Large numbers of marker-trait associations have been generated through QTL mapping studies (Collard and Mackill, 2008; Rey-Serra et al., 2021) which makes MAS a powerful tool for precision breeding. Implementation of high-throughput assays such as high-resolution melting (HRM) and simple sequence repeat analysis (SSR) paired with DNA markers and rapid high-throughput DNA extraction methods have enabled rapid improvement of octoploid strawberry varieties at the University of Florida (Noh et al., 2017). This success highlights the significant potential of MAS for varietal improvement of fruit crops.

Genomic selection (GS) is a method of selection which utilizes genome-wide variation and phenotypic data to predict phenotypes of an unobserved population (Bernardo, 1994; Meuwissen et al., 2001; Goddard and Hayes, 2007; Montesinos-López et al., 2021). GS offers the potential for increased genetic gain within a breeding program, as it enables increased selection intensity, selection accuracy, and reduction of the generational interval (Werner et al., 2023). Additionally, GS enables faster selection of clonally propagated crops such as those in the *Rosaceae* family by predicting their performance as clones while they are still in the seedling stage, allowing for earlier analysis of traits that would otherwise require further physiological development, such as fruit flavor and shelf life (Werner et al., 2023). Due to the complexity of the polyploid genome, generation of genome-wide SNP arrays can be complicated, contributing to the delay in adoption of GS methods for breeding of polyploid crops (Zingaretti et al., 2019). Despite the challenges posed by genomic complexity of polyploids, the continuous release of high-quality reference genomes has allowed for the parallel improvement of SNP genotyping methods. To aid in adoption of GS in polyploids, software such as polyploid Sequence Based Virtual Breeding (pSBVB) has been developed to simulate and evaluate GS strategies in polyploids and is equipped to simulate differences between allo- and autopolyploids (Zingaretti et al., 2019). Numerous reports have now been made for successful application of GS in octoploid strawberry (Gezan et al., 2017; Whitaker et al., 2017; Osorio et al., 2021). In a study by Yamamoto et al. (2021), 105 inbred strawberry lines were developed and used to train a GS model based on phenotyping data for petiole length, leaf area, Brix, fruit firmness, and pericarp color. Using the model to predict phenotypic values for a F1 hybrid testing population derived from these 105 lines revealed that phenotypic data collected from the parental inbred lines was sufficient to predict the F1 hybrid phenotypes when the model accuracy in cross-validation is sufficient (Yamamoto et al., 2021). Pincot et al. (2020) applied GS to strawberry to evaluate improve Verticillium wilt resistance. While the inclusion of wild genotypes in the training population reduced accuracy, the results suggested a strong potential for GS to identify superior resistant individuals if the model was sufficiently trained (Pincot et al., 2020).

Due to the wide range of research in strawberry, the volume of available genomic resources has increased significantly. As such, the Genome Database for Rosaceae (GDR) was created to house a wide range of tools and data. Among these tools is a compendium of strawberry DNA tests which can be downloaded from GDR^1^ and implemented in breeding programs (Oh et al., 2019).

### 2.1 Multi-omics approaches for trait discovery for improving strawberry

Enabled by the release of high-quality genome assemblies, genomic approaches have been applied to help identify candidate genomic regions and genes for several key strawberry traits, including flavor (Oh et al., 2021; Fan et al., 2022; 2023), disease resistance (Mangandi et al., 2017; Nellist et al., 2019; Nelson et al., 2021; Salinas et al., 2019), fruit firmness (Lee et al., 2021), fruit quality (Verma et al., 2017), and fruit shape (Nagamatsu et al., 2021). As genomic technologies continue to improve, they will allow for greater understanding of the genetic interactions and mechanisms underlying traits of breeding interest. QTL mapping has been performed in strawberry to identify loci underlying major quality and production traits such as day-neutrality, runner production, disease resistance, and fruit quality traits, among many others (Weebadde et al., 2008; Castro et al., 2015; Cockerton et al., 2018; Hossain et al., 2019; Alarfaj et al., 2021). Similarly, genome-wide association studies (GWAS) have been applied in strawberry for the discovery of numerous major traits (Pincot et al., 2018; Wada et al., 2020; Saiga et al., 2022). Recent advancements in genome sequencing technology have also enabled the analysis of large populations and generation of pangenomes, from which structural variants associated with key traits can be identified (Bohra et al., 2020). Pangenomics has been applied in strawberry to identify patterns in fruit color (Qiao et al., 2021). Analysis of strawberry pangenomes also resulted in interesting findings about strawberry evolution and domestication. Based on transposable element analysis of a pangenome constructed from 10 high-quality strawberry genomes, Lyu et al. (2023) suggested that *Fragaria viridis* may not be one of the diploid *Fragaria ×ananassa* ancestors as previously thought. Additionally, Qiao et al. (2021) discovered a new diploid strawberry species during assembly of their own pangenome. Discovery of transposable elements and other structural variants can be difficult when performing analysis of single genomes, however, such variants have been known to impact major agronomic traits (Tao et al., 2019), and are easier to identify through pangenome analysis. Thus, continued exploration of strawberry pangenomes may yield further insight into other major fruit quality and production traits and may assist in unravelling the complex evolutionary history of cultivated strawberry. Genomics-assisted technologies have also been widely implemented for trait discovery in crops outside of *Rosaceae*. New methods of QTL mapping have demonstrated capacity to resolve QTL candidates within a window of only a few kilobases (Bohra, 2013; Varshney et al., 2014; Zhang et al., 2019; Bohra et al., 2020), and in some cases, were able to generate QTL regions of which the resolution is comparable to the outcomes of sequence-based GWAS (Zhang et al., 2019; Bohra et al., 2020). Implementation of these new techniques in strawberry may enable further trait discovery for key agronomic traits.

In addition to the application of genomics for candidate gene identification, transcriptomics has been applied in strawberry to map expression quantitative trait loci (eQTLs) related to various fruit traits, including flavor (Sánchez-Sevilla et al., 2014; Barbey et al., 2020; 2021; Fan et al., 2022) disease resistance (Barbey et al., 2019), ripening and softening, and several others (Barbey et al., 2020). Strawberry transcriptome data has also recently been used to explore major postharvest issues, such as host responses to *Botrytis cinerea* infection (Yu et al., 2021) and differences in shelf life between cultivars (Min et al., 2020). In both cases, several candidate genes were identified which may be involved in plant defense, regulation of senescence, and shelf life (Min et al., 2020; Yu et al., 2021). Transcriptomic analyses of strawberry resulted in updates to existing genome annotations (Liu et al., 2021) and new transcriptome assemblies (Sánchez-Sevilla et al., 2017). Sufficient strawberry transcriptomic data has even been generated to allow for a meta-analysis of fruit ripening which resulted in the identification of previously unrevealed differentially expressed genes (DEGs) (Yi et al., 2021). Additionally, it is possible to identify allelic contributions based on relative expression patterns using transcriptomic data (Castillejo et al., 2020; Chandra et al., 2021; Oh et al., 2021). Considering this, transcriptomics could be a powerful tool for identifying candidates in polyploid crops such as strawberry, where unraveling the allelic contribution to a specific trait across homoeologous chromosomes remains challenging. As such, identification of transcriptionally dominant alleles may inform targeted trait improvement.

Despite the advancements in genome sequencing and transcriptomics which facilitate genome editing in octoploid strawberry, gene functional studies still rely heavily on transgenic approaches including RNA interference (RNAi) and overexpression (OE). Numerous traits, including several of postharvest interest, have been explored through transient and transgenic approaches in strawberry (Table 2). RNAi is an efficient tool for validating gene function through post-transcriptional gene silencing induced by double stranded RNA (Singh and Roychoudhury, 2023). RNAi and antisense approaches have also informed successful CRISPR/Cas strategies in strawberry (García-Gago et al., 2009; Paniagua et al., 2020; Paniagua et al., 2022; López-Casado et al., 2023). Transgenic application of tools like RNAi, antisense downregulation, and OE can be used to identify candidate genes prior to genome editing. Such transgenic lines depend on the continued expression of the recombinant constructs, which can vary depending on environmental or developmental effects. As such, these transgenic lines are not ideal to facilitate varietal improvement. Instead, identification and functionalization of candidate genes can be used to inform subsequent CRISPR/Cas genome editing. With the correct strategy, candidate genes can be knocked in or out, and alleles can be swapped for superior versions, allowing for altered gene expression or function and resulting phenotypes. The corresponding mutants can then be used in breeding schemes to further improve elite varieties. As such, transgenic approaches can be important tools to inform genome editing strategies for improvement of strawberry.

**TABLE 2:** Reported applications of transgenic approaches in strawberry for analysis of fruit quality and postharvest traits. (−) indicates value not reported.

Trait	Gene	Species	Variety	Method	Reference(s)
Flavor	*FaEGS*	*Fragaria x ananassa*	Calypso	Overexpression	Hoffmann et al. (2011)
	*FaIGS*	*Fragaria x ananassa*	Calypso	Overexpression	Hoffmann et al. (2011)
	*FaOMT*	*Fragaria x ananassa*	Elsanta	RNAi	Härtl et al. (2017)
	*FaF3H*	*Fragaria x ananassa*	Albion	RNAi	Jiang et al. (2013)
	*FaFAD1*	*Fragaria x ananassa*	(−)	RNAi	Oh et al. (2021)
Disease Resistance	*FaWRKY29*	*Fragaria x ananassa*	Florida Brilliance	RNAi	Lee et al. (2023)
	*FaWRKY64*	*Fragaria x ananassa*	Florida Brilliance	RNAi	Lee et al. (2023)
Fruit Size	*FaGAST2*	*Fragaria x ananassa*	Elsanta	RNAi; Overexpression	Moyano-Cañete et al. (2013)
Fruit ripening and softening	*FaSnRK2.6*	*Fragaria x ananassa*	Benihoppe	RNAi; Overexpression	Han et al. (2015)
	*njjs25*	*Fragaria x ananassa*	Chandler	Antisense downregulation	Jiménez-Bermúdez et al. (2002)
	*FaRIF*	*Fragaria x ananassa*	Camarosa	RNAi; Overexpression	Martín-Pizarro et al. (2021)
	*FaβGal4*	*Fragaria x ananassa*	Camarosa	Antisense downregulation	Paniagua et al. (2016)
	*FaPG1*	*Fragaria x ananassa*	Chandler	Antisense downregulation	Quesada et al. (2009)
		*Fragaria x ananassa*	Chandler	Antisense downregulation	Posé et al. (2013)
	*FaWRKY71*	*Fragaria x ananassa*	Benihoppe; Xiaobai	Overexpression	Yue et al. (2022)
	*FvPLA*	*Fragaria vesca*	Hawaii-4	RNAi; Overexpression	Zhang et al. (2022)
	*FaCHS*	*Fragaria x ananassa*	Elsanta	RNAi	Hoffmann et al. (2006)
		*Fragaria x ananassa*	Elsanta	Antisense downregulation	Lunkenbein et al. (2006)
		*Fragaria x ananassa*	Elsanta; Calypso	RNAi; Antisense downregulation	Hoffmann et al. (2011)
		*Fragaria x ananassa*	Sachinoka	RNAi	Miyawaki et al. (2012)
		*Fragaria x ananassa*	Elsanta	RNAi	Härtl et al. (2017)
	*FaPYR1*	*Fragaria x ananassa*	Fugilia	RNAi	Chai et al. (2011)
	*FaCTR1*	*Fragaria x ananassa*	Camarosa	RNAi	Sun et al. (2013)
	*FvWRKY48*	*Fragaria vesca*	Hawaii-4	RNAi; Overexpression	Zhang et al. (2022)
Fruit Color	*FvMYB10*	*Fragaria x ananassa*	Snow Princess	Overexpression	Wang et al. (2020a)
		*Fragaria vesca; Fragaria chiloensis*	WV596 (F. vesca); CS-52, FC285, FC156, FC157, FC160, FC157 (F. chiloensis)	Overexpression	Castillejo et al. (2020)
	*FaMYB1*	*Fragaria x ananassa*	Sachinoka	RNAi	Kadomura-Ishikawa et al. (2015)
	*FaDFR*	*Fragaria x ananassa*	Albion	RNAi	Lin et al. (2013)
	*FaANS*	*Fragaria x ananassa*	Calypso	Overexpression	Giampieri et al. (2018)
	*FvWRKY50*	*Fragaria x ananassa*	Benihoppe	RNAi; Overexpression	Chen et al. (2023)

Since metabolites are major contributors to fruit flavor and quality, it is necessary to identify genes associated with their production. Metabolomics can supplement transcriptomic and genomic data to support gene discovery on a biochemical level. In strawberry, metabolomics is frequently used in flavor studies (Schwab et al., 2008; Chambers et al., 2014; Fan et al., 2022), however, metabolomics has also been implemented in studies of plant stress response (Antunes et al., 2019), fruit development and ripening (Vallarino et al., 2018), and response to blue light (Chen et al., 2020). Like metabolomics, proteomics can be used in tandem with transcriptomic and genomic data to facilitate gene discovery. Application of proteomics and transcriptomics has been used to analyze postharvest quality changes during storage under different conditions, including controlled ozone treatments (Chen et al., 2019) and temperature stress (Lv et al., 2022). In both cases, comparisons of the differentially expressed proteins (DEPs) with the expression patterns of their respective genes found that proteosome responses mimicked the changes in postharvest quality, further supporting their proposed roles in stress response (Chen et al., 2019; Lv et al., 2022).

Multi-omics has been employed for trait discovery in strawberry and other crop species. In tomato, the correlation of SNPs, transcripts, and metabolites was used to identify new genes and pathways dictating major fruit traits (Zhu et al., 2018; Gaston et al., 2020). Similar application of multi-omics analysis in strawberry may discover novel pathways and gene candidates supporting fruit quality and production. If diverse evidence points to major genes for a desirable trait, CRISPR/Cas-mediated editing of the major gene or its repressor should have a high potential for crop improvement. As such, continual integration of genomics, transcriptomics, metabolomics, and proteomics is critical to inform CRISPR/Cas9 approaches for improvement of strawberry.

## 3 Genetic transformation and regeneration of strawberry

**FIGURE 1:**
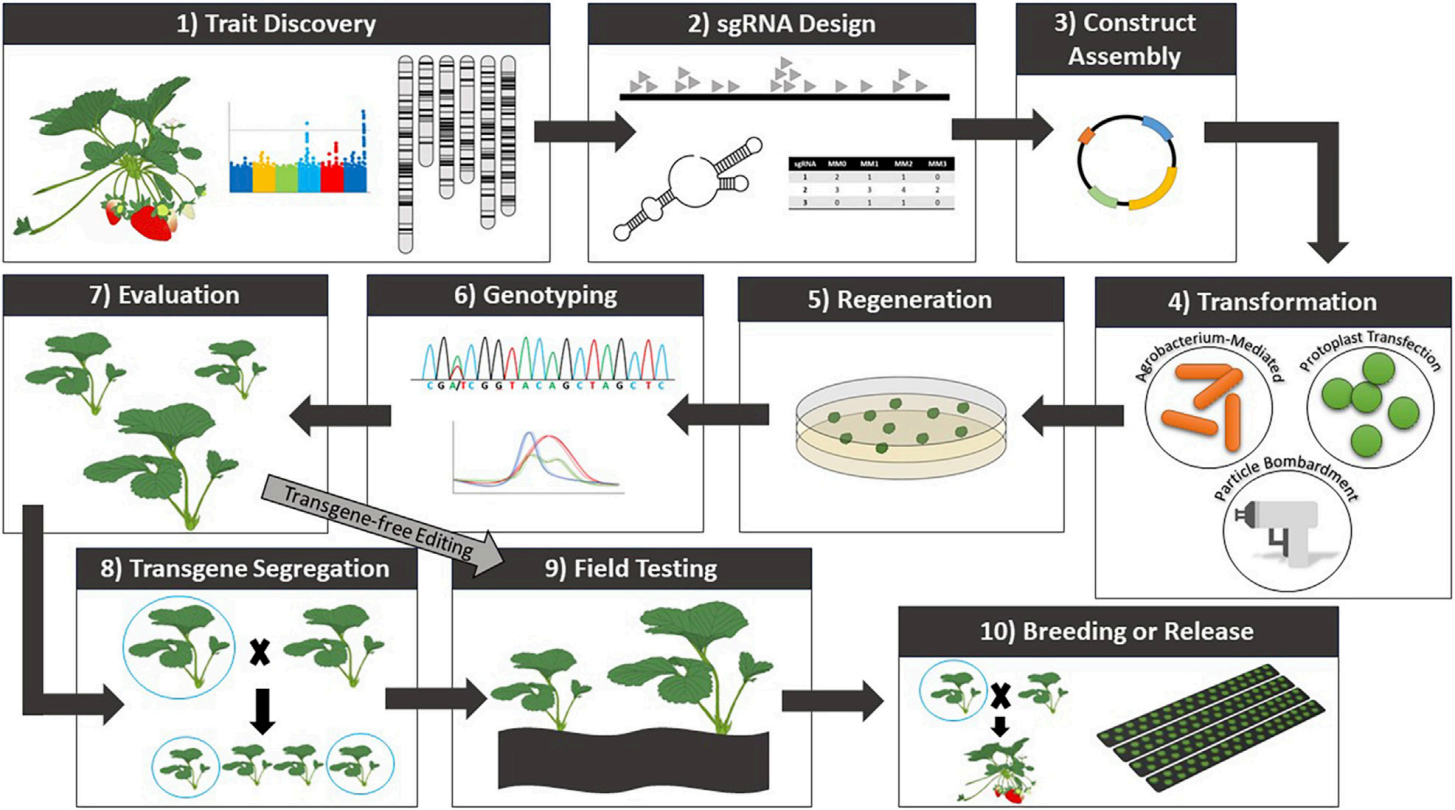
General workflow for trait discovery and CRISPR/Cas-mediated genome editing in strawberry.

Following trait discovery, genetic transformation can be performed to validate candidate gene function (Figure 1). While CRISPR/Cas-mediated genome editing has yet to be widely implemented and optimized for *Fragaria* species, numerous reports of transgenic modifications have been made. Agrobacterium-mediated transformation is currently the most applied transformation method for strawberry. Protocols using agrobacterium have undergone significant optimization to improve transformation efficiency. Thus far, protocols have been established for Agrobacterium-mediated transformation and subsequent regeneration of a range of tissues (Li et al., 2018a; Feng et al., 2019; Feng et al., 2021b; Wilson et al., 2019; Duan et al., 2021; Mao et al., 2022; Yan et al., 2023). In strawberry, agrobacterium-mediated transformation most commonly uses *Agrobacterium tumefaciens* strains LBA4404, GV3101, and MP90. Leaves are the most common explant material (Table 3). Transient methods of agrobacterium-mediated transformation have also been developed for fruit (Carvalho et al., 2016; Dai et al., 2020; Zeng et al., 2021; Mao et al., 2022; Lee et al., 2023) to study a range of mechanisms and traits, as well as to analyze the performance of DNA constructs prior to stable transformation. Particle bombardment (Agius et al., 2005) and protoplast transfection (Gou et al., 2020) have also been applied in strawberry for transient analyses. However, despite the demonstrated success of both methods in such analyses, stable transformation of strawberry with these approaches remains a challenge.

**TABLE 3:** Reported methods for genetic transformation of strawberry.

Transformation method	Details	Species	Genotype	Explant	Max. Transformation efficiency	Reference(s)
Agrobacterium	Agrobacterium tumefaciens strain MP90	*Fragaria x ananassa*	Redcoat	Leaf-derived callus	10/340 (2.9%)	Nehra et al. (1990a)
		*Fragaria x ananassa*	Redcoat	Leaf disks	6.50%	Nehra et al. (1990b)
	Agrobacterium tumefaciens strain LBA4404	*Fragaria x ananassa*	Festival; Sweet Charly	Leaf disks	10.8% (13/120) (Festival); 10.8% (13/120) (Sweet Charly)	Zakaria et al. (2014)
		*Fragaria x ananassa*	Chandler	Leaf disks (juvenile; adult)	4.16% (juvenile); 4.22% (adult)	Barceló et al. (1998)
		*Fragaria x ananassa*	Rapella	Leaf disks; petioles	6% (15/250) (Leaf disks); 1.45% (4/275) (petioles)	James et al. (1990)
		*Fragaria x ananassa*	Chandler	Leaf disks	12.60%	Quesada et al. (2009)
		*Fragaria x ananassa*	Induka; Elista	Leaf disks	4.5% (Induka); 9.5% (Elista)	Gruchała et al. (2004b)
		*Fragaria x ananassa*	Elista; Wega; Senga Precosa; Kama; Induka; Maria; Redgauntlet; Zao Hang Guang; Dukat; Favette; Vikat	Leaf disks	9.5% (Elista); 7.3% (Wega); 6.5% (Senga Precosa); 6.2% (Kama); 4.5% (Induka); 3.0% (Maria)	Gruchała et al. (2004a)
		*Fragaria x ananassa*	Symphony; Senga Sengana	Leaf disks	14.2% (Symphony); 2.7% (Senga Sengana)	Bachelier et al. (1997)
		*Fragaria x ananassa*	Rhapsody; Melody; Symphony	Stems	6%	Graham et al. (1995)
		*Fragaria x ananassa*	Tristar	Meristematic sections	13.6% (Tristar)	Mathews et al. (1995)
		*Fragaria vesca*	Hawaii-4; Reugen; Alexandria; accessions: 551552, 551782, 551791, 551792, 551833, 551890, 551892, 602578, 602923, 602931, 616513	Leaves	>100%	Oosumi et al. (2006)
		*Fragaria x ananasssa*	Pajaro	Leaf disks	6.60%	Vellicce et al. (2003)
		*Fragaria x ananassa*	Camarosa	Leaves	86.00%	Haddadi et al. (2015)
	Agrobacterium tumefaciens strain GV3101	*Fragaria x ananassa*	Shanghai Angel	Leaf-derived callus	5.3%	Duan et al. (2021)
		*Fragaria nilgerrensis*	Accession SN11-6	Leaf disks	8.67% (52/600)	Jiang et al. (2023)
		*Fragaria vesca*	PI 551572	Leaf disks	up to 89.7%	Pantazis et al. (2013)
	Agrobacterium tumefaciens strain CBE21	*Fragaria x ananassa*	Firework	Leaf disks	11.0%	Schestibratov and Dolgov (2005)
	Agrobacterium tumefaciens strain GV2260	*Fragaria x ananassa*	Gorella; Confitura; Chandler; Douglas; Brighton; Tioga; Senga Sengana; Addie; Athena; Fern	Leaf disks	Up to 4.43%	Husaini (2010)
	Agrobacterium tumefaciens strain EHA105	*Fragaria vesca; Fragaria x ananassa*	Alpine (accession FRA197; accession FRA198); Hecker; La Sans Rivale	Leaves/petioles	64.4% (47/73) (FRA197); 67.9% (36/53) (FRA198); 10.4% (5/48) (Hecker); 7.4% (4/54) (La Sans Rivale)	Zhao et al. (2004)
		*Fragaria x ananassa*	Totem	Leaves/petioles	15.6% (Totem)	Mathews et al. (1995)
	Agrobacterium tumefaciens strain EHA101	*Fragaria x ananassa*	Tristar; Totem	Meristematic sections (Tristar); leaves/petioles (Totem)	16.7% (Tristar); 58.8% (Totem)	Mathews et al. (1995)
	Agrobacterium tumefaciens strain AGL0	*Fragaria x ananassa*	Calypso	Leaf disks	100%	Schaart (2014)
		*Fragaria x ananassa*	Elsanta	Fruit	100%	Hoffmann et al. (2006)
	Agrobacterium rhizogenes strains Ar1193; K599; C58C1; MSU440	*Fragaria vesca*	Yellow Wonder (5AF7)	Cotyledons; hypocotyls; SC; leaves; petiols	71.43%	Yan et al. (2023)
Particle Bombardment	Gold nanoparticles (1.6 µm) coated with Agrobacterium tumefaciens strain LBA4404	*Fragaria x ananassa*	Chandler	Leaf disks	21%	Cordero de Mesa et al. (2000)
	Gold nanoparticles (0.6 µm) coated with viral RNA	*Fragaria x ananassa*	Yotsuboshi; Dover	Leaves	100% (Yotsuboshi); 100% (Dover)	Li et al. (2019a)
	Tungsten particles coated with plasmid	*Fragaria x ananassa*	Toyonaka	Anther-derived callus	15.40%	Wang et al. (2004)
Protoplast	PEG-mediated transfection	*Fragaria vesca*	Hawaii	Leaf-derived protoplasts	60%	Gou et al. (2020)
	Protoplast electroporation	*Fragaria x ananassa*	77101	Leaf/petiole-derived protoplasts	5 × 10^−4^	Nyman and Wallin (1992b)

Transformation of strawberry has also been performed using the hairy root system (Yan et al., 2023). Hairy roots are valuable for functional analysis of root traits as well as for validation of transgenic and genome editing methods due to the relatively short period of root development ​​(Ozyigit et al., 2013)​. Several advancements have been made to Agrobacterium-mediated transformation technologies, including improved ternary systems (Anand et al., 2018), auxotrophy of various amino acids (Aliu et al., 2020; Prías-Blanco et al., 2022), and use of a CRISPR RNA-guided integrase system (Vo et al., 2021; Aliu et al., 2022), however, there has been little application of these advanced systems in strawberry (Oosumi et al., 2006).

Some transgene-free methods, such as ribonucleoprotein (RNP) bombardment and RNP transfection of protoplasts, have not been reported in strawberry but have demonstrated success in a range of crops (Liu et al., 2020; Zhang et al., 2021a; Najafi et al., 2023). Other methods of transformation and editing, such as RNA bombardment (Li et al., 2019a) and virus-mediated transformation (Tian et al., 2015) have been sporadically applied in strawberry, indicating a need for further development. Transgene-free targeted mutagenesis approaches for varietal development fall within improved consumer acceptance and reduced regulatory constraints. Thus, continued refinements of genomic tools, transformation and genome editing strategies will position CRISPR/Cas technology as primary tool for gene function validation and crop improvement.

Further development of efficient protoplast isolation, transformation, and regeneration is also important for future transgene-free editing of strawberry. Isolation and regeneration of protoplasts is well established for strawberry (Nyman and Wallin, 1988; 1992a; Barceló et al., 2019; Gou et al., 2020). However, while protocols have been established for transient analyses in protoplasts, few reports of successful regeneration involve transformed materials (Nyman and Wallin, 1992b; Pattanaik et al., 2004; Gou et al., 2020). As such, it is necessary to continue developing methods to transform and regenerate plants from strawberry protoplasts as a foundation for transgene-free genome editing.

As has been established in other species, transformation and regeneration of strawberry depends on several factors, and protocol optimization can be challenging. Experiments to optimize strawberry transformation have observed a wide range of transformation and regeneration efficiencies which vary significantly between genotypes (Zakaria et al., 2014). Other factors, such as transformation method and explant material, have also been reported to impact regeneration efficiency, and response to these factors also varies strongly by genotype (Table 3). Taken together, these findings suggest that some genotype-specific optimization of protocols will be necessary for efficient genetic transformation. Additionally, transformation and regeneration efficiencies tend to be higher for diploid strawberry than octoploid strawberry, even when other factors are held constant between species (Table 3). As *Fragaria ×ananassa* is the species of economic interest, continued optimization to improve both transformation and regeneration efficiencies will be essential to facilitate genome editing for varietal improvement.

## 4 Recent advances and resources in CRISPR/Cas-mediated genome editing in strawberry

CRISPR, or Clustered Regularly Interspaced Palindromic Repeats, is a genome editing system derived from a bacterial defense network. In bacteria, this defense network operates in two phases to incorporate short fragments of invading DNA into the bacterial genome and then use these sequences to recognize and cleave foreign DNA based on the presence of a protospacer adjacent motif (PAM) (Doudna and Charpentier, 2014; Vigouroux and Bikard, 2020). For genome editing via CRISPR/Cas systems, this bacterial defense pathway is manipulated to target specific sequences within a genome of interest. Unlike other methods of genome editing, such as zinc-finger nucleases (ZFNs) and transcription activator-like nucleases (TALENs), which require substantial protein engineering, CRSIPR/Cas genome editing can be performed simply through a change in the single guide RNA (sgRNA) sequence (Doudna and Charpentier, 2014). The ability to switch editing targets quickly without need for protein engineering has played a major role in the rise of popularity of CRISPR/Cas genome engineering. Double stranded breaks (DSBs) are generated when a Cas endonuclease cleaves DNA at a targeted site using a sgRNA as reference (Cong et al., 2013; Mali et al., 2013; Doudna and Charpentier, 2014). These DSBs can then be repaired through non-homologous end joining (NHEJ), or template mediated homology-directed repair (HDR). NHEJ is error prone, often resulting in insertions or deletions that cause loss of gene function (Cong et al., 2013; Mali et al., 2013; Chen et al., 2022). Targeted mutagenesis using NHEJ mediated repair of CRISPR/Cas-mediated DSBs has been reported in many Rosaceous crops, including apple (Malnoy et al., 2016; Pompili et al., 2020), pear (Charrier et al., 2019; Pang et al., 2019), raspberry (Miller, 2019), and strawberry (Martín-Pizarro et al., 2021; López-Casado et al., 2023). In contrast, template mediated HDR allows precise conversion of targeted single nucleotides or insertion of a specific sequence. Allelic variants differing in single-nucleotide polymorphisms often confer improvement of agronomic traits. HDR pathways can be leveraged to replace alleles with superior variants and has successfully been implemented in crops such as maize, rice, and sugarcane (Shi et al., 2017; Wang et al., 2017; Oz et al., 2021). Reports of HDR-mediated gene targeting are still lacking in strawberry, likely due to low efficiency caused by infrequent occurrence of HDR, competition with NHEJ for DSB repair, and inadequate repair template in close proximity to the DSB site (Chen et al., 2022).

Similar to template mediated HDR, both base and prime editing can generate precision nucleotide substitutions in target genes. Base editing occurs as the result of a catalytically impaired Cas nuclease, such as Cas nickase (nCas) or dead Cas (dCas), fused to a nucleotide deaminase (Molla et al., 2021) and results in an irreversible base conversion without the need for DSBs or an exogenous template (Azameti and Dauda, 2021). Base editing requires the use of different deaminases depending on the desired nucleotide substitution, is currently limited to six of the 12 possible base-swaps, and may result in bystander mutations (Molla et al., 2021). In contrast, prime editing, which occurs as the result of fusing a nCas nuclease with a reverse transcriptase, is capable of generating all 12 possible substitutions as well as small indels in exchange for lower editing efficiency (Molla et al., 2021). Base editing has been applied for the creation of precision nucleotide substitutions in strawberry to support the fine tuning of the sugar content of the strawberry fruit (Xing et al., 2020). While prime editing has not been reported in strawberry, it has been successfully applied in tomato, rice, and wheat (Lin et al., 2020; Xing et al., 2023), demonstrating its potential for precision nucleotide substitution in plant systems.

In addition to generating nucleotide substitutions through base or prime editing, CRISPR/Cas can also be applied to modulate gene expression patterns and epigenetic regulation. By fusing dCas with different effector proteins, it is possible to achieve efficient targeted activation (CRISPRa), repression (CRISPRi), or epigenome modifications (Pan et al., 2021).

While genome editing using the CRISPR/Cas system has largely focused on the use of the Cas9 endonuclease, additional Cas nucleases have been engineered to improve the flexibility of the CRISPR genome editing system by relaxing the requirements for a specific protospacer adjacent motif and altering nuclease function (Guilinger et al., 2014; Shen et al., 2014; Trevino and Zhang, 2014; Tsai et al., 2014; Anders et al., 2016; Komor et al., 2016; 2017; Havlicek et al., 2017; Harrington et al., 2018; Liu et al., 2019; Anzalone et al., 2020; Ghogare et al., 2020; Zhang et al., 2021b; Sukegawa et al., 2023). This is particularly useful for generation of precision nucleotide substitutions using base or prime editing (Wang et al., 2020b; Kantor et al., 2020; Mishra et al., 2020; Huang and Puchta, 2021).

Genome editing is a powerful tool for crop improvement, as it allows for precise, targeted mutation in one or few genes without altering the plant’s genetic background. Genome editing can be an efficient method for varietal improvement as co-editing of multiple genes or alleles by multiplex editing allows researchers to accelerate the generation of desired combinations in elite germplasm without undergoing meiotic recombination (El-Mounadi et al., 2020). In contrast, conventional breeding schemes typically require numerous generations and backcrossing to improve gene or allele combinations for a single trait of interest. Genome editing with CRISPR/Cas also enables the introduction of traits that do not exist within a breeding germplasm. For example, there are currently no widely available breeding sources of resistance to *Botrytis cinerea* in strawberry, and previous breeding efforts to increase resistance to Botrytis fruit rot (BFR) have been ineffective (Petrasch et al., 2019). However, substantial research has been performed to identify susceptibility genes related to BFR and other strawberry diseases which could make for useful knockout targets in the future.

### 4.1 Challenges for genome editing in octoploid strawberry

While the potential benefits of CRISPR/Cas genome editing in *Fragaria ×ananassa* are numerous, there are several challenges which must be overcome. Octoploid strawberries are highly heterozygous as compared to diploid strawberries (Martín-Pizarro et al., 2019), which can make target gene identification and design of efficient sgRNAs difficult (May et al., 2023). Additionally, the four homoeologous subgenomes of *Fragaria ×ananassa* are not separated from each other, rather, they have undergone numerous homoeologous exchanges which resulted in increased genomic complexity (Whitaker et al., 2020).

Traditionally, transgene-free genome edited plants are generated through sexual segregation, which is often a labor-intensive and time-consuming process (Gao, 2021). Cultivated strawberries are asexually propagated hybrids, meaning segregation of transgene-free plants through segregation is often impractical. Instead, it is necessary to continue developing other transgene-free genome editing methods for the improvement of strawberry. In addition to optimizing transformation procedures, further development of systems such as the transgene killer CRISPR (TKC), which is able to automatically self-destruct the transgene through inclusion of suicide genes in the CRISPR/Cas construct (He et al., 2018; He et al., 2019; Gu et al., 2021), may enable transgene-free genome editing of strawberry. To date several studies for transgene-free genome editing of Rosaceous crops have been performed (Malnoy et al., 2016; Osakabe et al., 2018; Pompili et al., 2020), though regeneration of explants and selection of transgene-free plants remains a challenge.

### 4.2 Target traits for CRISPR genome editing in cultivated strawberry (*F*. ×*ananassa*)

**FIGURE 2:**
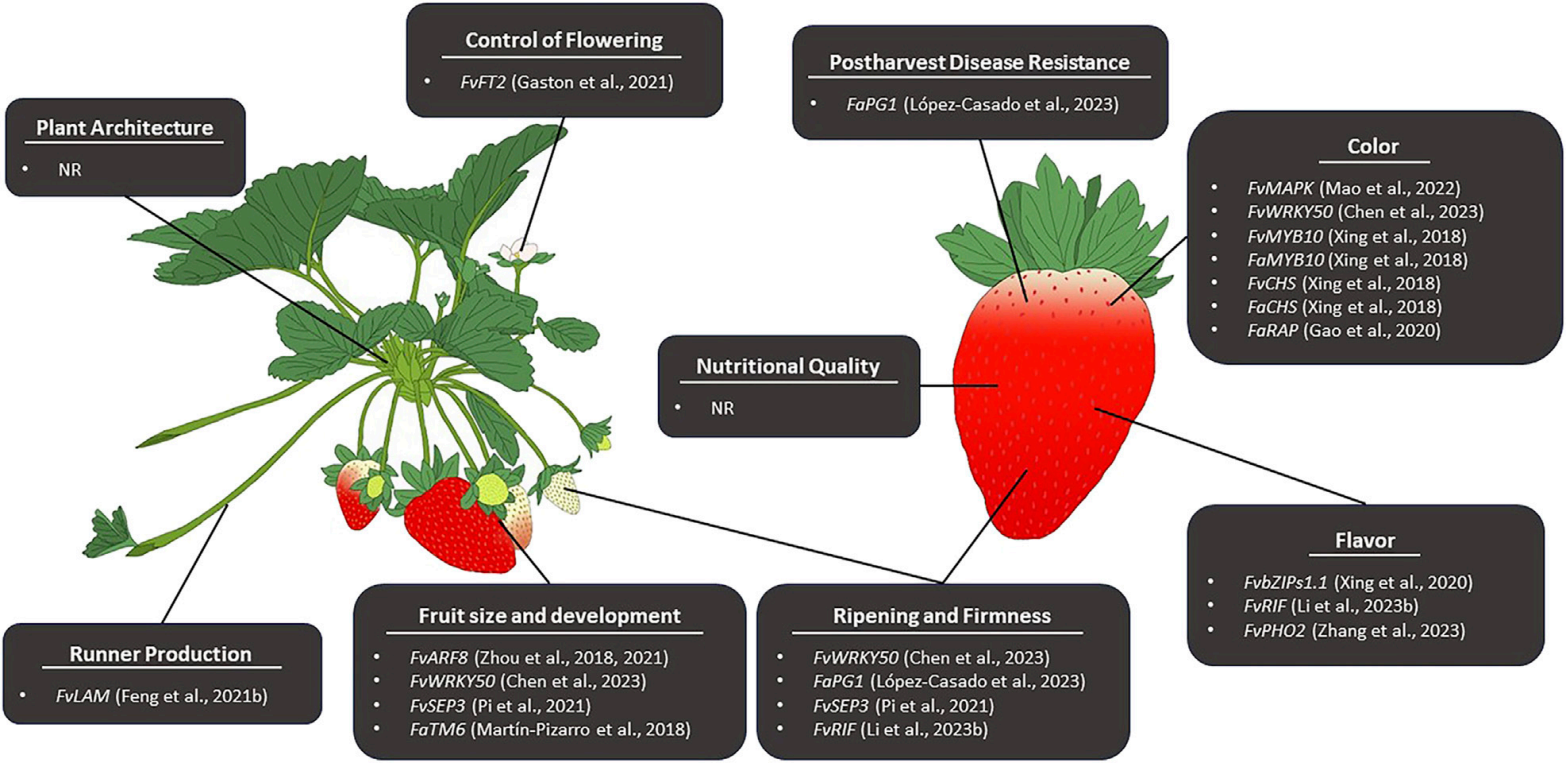
Reported CRISPR/Cas-mediated editing for the improvement of pre- and postharvest traits associated with strawberry fruit quality and production. NR indicates no genome editing has been reported to affect a given trait.

Genome editing in strawberry can be divided into two themes; editing performed in the diploid strawberry and editing performed in the octoploid strawberry. Genome editing is more commonly performed in diploid strawberry due to the simple nature of its genome and its status as a model system for *Rosaceae*. In the diploid strawberry, genome editing has been successfully employed to manipulate numerous traits (Table 4). Until recently, the complexity of the octoploid genome posed a significant challenge to CRISPR/Cas genome editing, and as such, far less exploration of genome editing in *Fragaria ×ananassa* has been performed. Due to the availability of new, high-quality octoploid genome assemblies, genome editing has recently been applied for the improvement of several traits (Table 4; Figure 2) and may become a powerful tool for trait discovery and gene characterization in cultivated strawberry.

**TABLE 4:** Reported CRISPR/Cas-mediated genome editing in strawberry.

Trait	Gene(s)	Species	Transformation method	Mutant Phenotype(s)	Reference(s)
Runner Production	*FvLAM*	*Fragaria vesca*	Agrobacterium (strain GV3103)	Significant reduction of runner production	Feng et al. (2021b)
Fruit Color	*FvMAPK*	*Fragaria vesca*	Agrobacterium (strain EHA105)	Increased anthocyanin accumulation at low temperatures	Mao et al. (2022)
	*FvWRKY50*	*Fragaria vesca*	Agrobacterium	Delayed anthocyanin accumulation	Chen et al. (2023)
	*FvCHS/FaCHS*	*Fragaria vesca, Fragaria x ananassa*	Agroinfiltration of fruit	Partial delay of anthocyanin accumulation in Fragaria vesca; no observed effects in Fragaria x ananassa	Xing et al. (2018)
	*FvMYB10/FaMYB10*	*Fragaria vesca, Fragaria x ananassa*	Agroinfiltration of fruit	Partial delay of anthocyanin accumulation in Fragaria vesca; no observed effects in Fragaria x ananassa	Xing et al. (2018)
	*FaRAP*	*Fragaria vesca, Fragaria x ananassa*	Agrobacterium (strain GV3101)	White skin and flesh; reduction in total anthocyanin content	Gao et al. (2020)
Carotenoid Biosynthesis	*FvPDS*	*Fragaria vesca*	Stable transformation	Albino and chimeric photobleached plants	Xing et al. (2018)
		*Fragaria vesca, Fragaria x ananassa*	Agrobacterium (strain EHA105)	Albino and variegated tissue	Wilson et al. (2019)
Flower and Fruit Development	*FvARF8*	*Fragaria vesca*	Agrobacterium	Increased fruit size	Zhou et al. (2021)
	*FvGID1C*	*Fragaria vesca*	Agrobacterium	Severe retardation of plant growth; lack of flowering shoots	Zhou et al. (2021)
	*FvWRKY50*	*Fragaria vesca*	Agrobacterium	Early flowering; malformed fruits (one line)	Chen et al. (2023)
	*FvSEP3*	*Fragaria vesca*	Agrobacterium	Aberrant flower formation; failure of fertilization; parthenocarpic fruit development	Pi et al. (2021)
	*FvMAPK*	*Fragaria vesca*	Agrobacterium (strain EHA105)	Oblate fruits	Mao et al. (2022)
	*FvLAM*	*Fragaria vesca*	Agrobacterium (strain GV3103)	Flowers lacked stamens or developed very few stamens	Feng et al. (2021b)
	*FaPG1*	*Fragaria x ananassa*	Agrobacterium (strain AGL1)	Reduced fresh fruit weight; altered fruit shape	López-Casado et al. (2023)
	*FaTM6*	*Fragaria x ananassa*	Agrobacterium (strain LBA4404)	Abnormal flower morphology; decreased pollen production; arrested receptacle development	Martín-Pizarro et al. (2019)
Control of Flowering	*FvFT2*	*Fragaria vesca*	Agrobacterium (strain GV3101 or C58C1)	Delayed flowering	Gaston et al. (2021)
Fruit Ripening and Firmness	*FvWRKY50*	*Fragaria vesca*	Agrobacterium	Delayed anthocyanin accumulation	Chen et al. (2023)
	*FvSEP3*	*Fragaria vesca*	Agrobacterium	Delayed fruit ripening	Pi et al. (2021)
	*FvRIF*	*Fragaira vesca*	Agrobacterium (strain GV3101)	Inhibition of ripening; decreased anthocyanin content; inhibited fruit softening	Li et al. (2023b)
	*FaPG1*	*Fragaria x ananassa*	Agrobacterium (strain AGL1)	Significant increase in fruit firmness	López-Casado et al. (2023)
Abiotic Stress Tolerance	*FvICE1*	*Fragaria vesca*	Agrobacterium (strain GV3101)	Decreased tolerance to cold and drought stress	Han et al. (2023)
	*FvMYB46*	*Fragaria vesca*	Agrobacterium	Reduced osmotic stress resistance	Bjorå et al. (2021)
	*FaPG1*	*Fragaria x ananassa*	Agrobacterium (strain AGL1)	Reduced surface area of Botrytis cinerea infection	López-Casado et al. (2023)
Auxin Signaling and Biosynthesis	*FvWRKY50*	*Fragaria vesca*	Agrobacterium	Dwarf phenotype (one line); early initiation of leaf senescence; significant reduction in auxin content	Chen et al. (2023)
	*FvTAA1*	*Fragaria vesca*	Agrobacterium	No obvious morphological changes observed	Zhou et al. (2018)
	*FvARF8*	*Fragaria vesca*	Agrobacterium	Increased sensitivity to auxin and gibberellic acid; faster seedling growth; increased seedling size	Zhou et al. (2021)
	*FvYUC10*	*Fragaria vesca*	Agrobacterium (strain GV3101)	No obvious morphological changes observed; reduced free auxin content in fruit	Feng et al. (2019)
Fruit Flavor/Quality	*FvbZIPs1.1*	*Fragaria vesca*	Agrobacterium (strain EHA105)	Increased sugar content in T1 lines	Xing et al. (2020)
	*FvRIF*	*Fragaira vesca*	Agrobacterium (strain GV3101)	Decreased sugar content	Li et al. (2023b)
	*FvPHO2*	*Fragaria vesca*	Agrobacterium (strain GV3101)	Increased phosphorous content in fruits and leaves; dwarf growth habit; increased fruit anthocyanin content; increased fruit soluble solids content	Zhang et al. (2023)

#### 4.2.1 Manipulation of runner production to increase fruit production

In strawberry, differentiation of runners and branch crowns is mutually exclusive and is influenced by a range of environmental factors (Thompson and Guttridge, 1959; Hytönen et al., 2009; Mouhu et al., 2013; Tenreira et al., 2017; Caruana et al., 2018) However, since each plant produces a limited number of axillary meristems, runner production is considered inversely proportional to fruit production (Tenreira et al., 2017). For this reason, runner removal is a common cultural practice and has demonstrated positive impacts on both fruit quality and yield (Sønsteby et al., 2021). Many studies have attempted to characterize the relationship between fruit and runner production through manipulation of environmental conditions and plant hormones (Hartmann, 1947; Thompson and Guttridge, 1959; Mouhu et al., 2013; Qiu et al., 2019).

As genomic resources became more widely available, studies began to focus on the genes which dictate the decision of flowering versus runnering in strawberry. *Suppressor of Overexpression of Constans1* (*FvSOC1*), *FvGA20ox4, FvRGA1,* and *Loss of Axillary Meristems* (*FvLAM*) have all been identified as runner-associated genes (Mouhu et al., 2013; Tenreira et al., 2017; Caruana et al., 2018; Hytönen and Kurokura, 2020; Feng et al., 2021b). All runner-associated genes which have been identified to date are related to gibberellic acid biosynthesis, suggesting that gibberellic acid plays a major role in the decision between flowering and runnering. The full pathway of gibberellic acid biosynthesis in strawberry remains to be elucidated, however the proposed pathway has undergone continuous expansion as new runnering and flowering-associated genes are identified.

Despite the identification of numerous genes associated with runner production, so far CRISPR/Cas9 genome editing has only been reported in *FvLAM* in *Fragaria vesca* (Feng et al., 2021b), and no reports of genome editing of runner-associated genes in *Fragaria ×ananassa* have been made. CRISPR/Cas9 genome editing has also been applied in tomato and potato for the manipulation of similar traits (Zsögön et al., 2018; Cui et al., 2020; Tang et al., 2022; Tuncel and Qi, 2022), indicating that genome editing can be used effectively to alter plant growth habits. Plant architecture has been shown to have major impacts on yield in numerous crops (Sakamoto and Matsuoka, 2004; Srivastava et al., 2019), making the trait a prime candidate for manipulation via genome editing.

#### 4.2.2 Plant architecture and control of flowering

The timing and duration of flowering play pivotal roles in determining yield potential and harvest season length (Koskela et al., 2012). Manipulation of daylength sensitivity can enable earlier harvest (Soyk et al., 2017) and can even lead to multiple harvests within a single season (de Camacaro et al., 2002; Hancock et al., 2008). During the domestication of strawberry, development of perpetual flowering through photoperiod insensitivity was essential for extending both the range and production period (Gaston et al., 2021). Increased branching can also support increased inflorescences, enabling a greater yield per plant (Premsekhar and Rajashree, 2009). Since the axillary meristem-derived branch crowns are the primary bearer of inflorescences in strawberry, it may be possible to increase fruit yield by increasing floral branching.

*Terminal Flower1* (*FvTFL1*), *Flowering Locus T* (*FvFT2* and *FvFT3*), and *FvWRKY50* were identified as flowering-related genes and shown to interact with each other to control photoperiod response and flowering architecture (Iwata et al., 2012; Koskela et al., 2012; Mouhu et al., 2013; Gaston et al., 2021; Chen et al., 2023). While timing, duration, and development of flowers are important considerations for fruit production, reports of successful editing have been limited to diploid strawberry. *FvFT2* and *FvWRKY50* have undergone successful genome editing (Gaston et al., 2021), however, editing of other strawberry flowering-related genes has not been reported. Several genes have been identified in other crops with respect to plant architecture and flowering. *Branched 1* (*AtBRC1*), *Branched 2* (*AtBRC2*), *SlBRC1b, AtMAX1*, *AtMAX2*, *AtCYP79*, and *AtSPS* have been identified to negatively affect branching (Reintanz et al., 2001; Tantikanjana et al., 2001; Stirnberg et al., 2002; Greb et al., 2003; Aguilar-Martínez et al., 2007; Finlayson, 2007; Martín-Trillo et al., 2011). Similarly, *Self Pruning 5g* (*SlSP5G*) and *Flowering Locus C* (*BrFLC2*) resulted in early flowering and daylight insensitivity (Soyk et al., 2017; Jeong et al., 2019). As no editing of plant architecture or flowering-associated genes has been reported in octoploid strawberry, further functional analysis of known genes is necessary for the improvement of existing varieties.

#### 4.2.3 Fruit development

Further study of fruit development genes in strawberry may also enable improvement of fruit production. Parthenocarpy may enable more consistent yields under variable conditions (Hu et al., 2023), and larger fruits tend to be easier to harvest and more desirable to consumers (Hortyński et al., 1991). Both auxin and gibberellic acid have been well established as major regulators of strawberry fruit development (Sharma and Singh, 2009; Liu et al., 2012; Kang et al., 2013; Feng et al., 2019; Fuentes et al., 2019; Zhao et al., 2021) though morphological and environmental factors are also major contributors (Hortyński et al., 1991; Mackenzie et al., 2011; Menzel, 2019; 2021). Through domestication, the average strawberry fruit weight has increased from 1 to 3 g (*Fragaria virginiana*) to more than 20 g (*Fragaria ×ananassa*) (Chandler et al., 2012).

Knockout of *FvYUC10* altered patterns of auxin accumulation in fruit but resulted in no obvious morphological changes (Feng et al., 2019). RNAi of *FvYUC6* was found to negatively affect fruit development (Liu et al., 2014). Other *YUC* family genes have been identified through transcriptomic studies of strawberry fruit development (Liu et al., 2012; Feng et al., 2019) but have not undergone additional functionalization. Mutations in *FvRGA1* and genome editing in *Sepallata 3* (*FvSEP3*) resulted in parthenocarpic fruit development. Genome editing in *FvARF8* resulted in increased fruit sizes (Pi et al., 2021; Zhou et al., 2021) and RNAi of *FvYUC6* and *FveCYP707A4a* resulted in reduced fruit sizes (Liu et al., 2014; Liao et al., 2018). Reduction of fruit size was also accomplished through overexpression of *Gibberellin Stimulated Transcript* (*FaGAST1 and FaGAST2*) (de la Fuente et al., 2006; Moyano-Cañete et al., 2013). Knockout of *Tomato MADS box gene6* (*TM6*) in both diploid and octoploid strawberry resulted in defects in the anthers and arrested development of the receptacle (Martín-Pizarro et al., 2019), indicating that *TM6* also plays a major role in development of both strawberry flowers and fruits.

Significant work has also been performed to explore the mechanisms underlying fruit development in other crops. In apple, a strong QTL for fruit weight was linked to *Auxin Response Factor 106* (*MdARF106*) (Devoghalaere et al., 2012). Silencing of *SlIAA7* in tomato resulted in thicker pericarp tissue, and thus, larger fruits (Su et al., 2014). Simultaneous knockout of *SlARF8a* and *SlARF8b* resulted in parthenocarpic fruit development and increased parthenocarpic fruit sizes (Hu et al., 2023), and knockout of *Fasciated* (*SlFAS/SlCLV3*), *Fruit Weight 2.2* (*SlFW2.2*), and *Excessive Number of Floral Organs* (*SlENO*) resulted in increased locule numbers and larger fruits (Zsögön et al., 2018; Yuste-Lisbona et al., 2020). In addition to these genes, some studies have explored the impacts of hormone pathways on fruit development. These studies found that application of gibberellic acid at low concentrations positively impacted speed of development and yield, and decreased production of malformed fruits (Turner, 1963; Sharma and Singh, 2009; Jamal Uddin et al., 2012), further supporting the potential to improve fruit production through manipulation of hormone biosynthesis and signaling.

#### 4.2.4 Fruit flavor

Strawberry aroma is the result of a complex mixture of more than 360 volatile compounds (Zabetakis and Holden, 1997; Zorrilla-Fontanesi et al., 2012; Liu et al., 2023), however, only around six odor-active compounds which significantly contribute to flavor have been identified in cultivated strawberry (Raab et al., 2006; Ulrich et al., 2007; Hoffmann et al., 2011). As in many other fruit species, early breeding efforts in strawberry focused on improving firmness and other morphological traits at the expense of flavor and aroma (Hoffmann et al., 2011). As such, new efforts are underway to improve strawberry flavor and aroma.

Several genes associated with strawberry flavor and sugar content have been identified through QTL mapping, genome-wide association studies, and transcriptomic studies (Raab et al., 2006; Chambers et al., 2012; Zorrilla-Fontanesi et al., 2012; Shanmugam et al., 2017; Jiu et al., 2018; Lee et al., 2018; Porter et al., 2023). RNAi of strawberry *Chalcone Synthase* (*FaCHS*) paired with overexpression of either *Eugenol Synthase* (*FaEGS*) or *Isoeugenol Synthase* (*FaIGS*) resulted in partial restoration of wild strawberry aroma (Hoffmann et al., 2011). Additionally, RNAi of *Anthranilic Acid Methyl Transferase* (*FaAAMT*), *Anthranilate Synthase Alpha Subunit 1* (*FaASa1*), *FaFAD1,* and *FaTM9* resulted in changes in volatile profiles and soluble solids content (Pillet et al., 2017; Vallarino et al., 2019; Oh et al., 2021; Fan et al., 2022). Overexpression of *FaOMT* resulted in increased levels of mesifurane, another key volatile compound in strawberry (Fan et al., 2022). Base editing has also been successfully applied to modify fruit sugar content. Xing et al. (2020) used the A3A-PBE base editor to target the conserved sucrose control uORF of *FvbZIPs1.1*, resulting in 35 novel genotypes that displayed a range of sugar contents.

While flavor is a primary focus of varietal improvement in cultivated strawberry, there are no reports of successful editing of flavor genes to date. However, CRISPR/Cas has been applied in several fruit crops for flavor improvement. Knockout of *SlINVIVH1* and *SlVPE5* in tomato resulted in increases in sugar content and total soluble solids content in single and double mutant lines (Wang et al., 2021a). Genome editing has also been applied for the improvement of flavor traits in vegetables. Karlson et al. (2022) reported successful application of CRISPR/Cas12a to reduce pungency in *Brassica juncea*, resulting in increased consumer appeal without reducing nutritional content. This work was performed within the company Pairwise, in Durham, North Carolina, and salad mixes composed of the edited *Brassica juncea* plants have recently been commercially released (Brown, 2023), indicating commercial potential for genome edited crops with improved flavor traits. These successes highlight the potential applications of genome editing technology for the improvement of flavor in strawberry.

#### 4.2.5 Fruit color

Consumer preferences of fruit color can vary significantly across the globe (Whitaker et al., 2020), and as such, strawberries are available in a wide range of colors. Strawberry coloration is primarily due to variation in accumulation of anthocyanin in the receptacle and achenes during ripening. As fruit color is an important fruit quality trait for consumers, it is a common focus of selection in breeding programs and has undergone substantial investigation to identify associated genes.

Natural mutations in *FaMYB10* have been reported as the only natural sources of color variation in strawberry (Castillejo et al., 2020). These findings are supported by those of others, which have identified significant roles of *MYB10* and other *MYB* family genes in controlling anthocyanin accumulation and biosynthesis in strawberry (Whitaker et al., 2020; Denoyes et al., 2023). However, other genes have been reported to impact fruit color in addition to *MYB10*. Overexpression of *FvMYB10* and *Reduced Anthocyanins in Petioles* (*FvRAP*) resulted in restoration of anthocyanin biosynthesis in white fruits (Castillejo et al., 2020; Gao et al., 2020). While there is agreement that *FvRAP* plays a role in regulation of anthocyanin accumulation in addition to *FvMYB10*, conflicting conclusions have been reached regarding the position of *FvRAP* in the pathway. Luo et al. (2018) suggested that *FvRAP* operates downstream of and may be regulated by *FvMYB10*, whereas Gao et al. (2020) suggested that *FvRAP* may participate in a color development mechanism separate from *FvMYB10*. Xing et al. (2018) attempted CRISPR/Cas9 genome editing of *FvMYB10* and *FvCHS* in strawberry through an agroinfiltration of diploid and octoploid fruits but observed no noticeable delay in anthocyanin accumulation in octoploid fruits and only partial delay in diploid fruits. Knockout of *FvWRKY50* resulted in downregulation of several anthocyanin-associated genes, including *FvMYB10*, in addition to delayed anthocyanin accumulation (Chen et al., 2023), and knockout of *FvMAPK3* resulted in similar rates of anthocyanin accumulation but higher total anthocyanin content than empty vector controls (Mao et al., 2022).

Genome editing for fruit color modification has also been implemented in other species. In tomato, CRISPR/Cas9-mediated genome editing was used to generate tomatoes that were yellow, pink, and purple in color (Čermák et al., 2015; Filler Hayut et al., 2017; Deng et al., 2018), indicating the potential to fine tune fruit color through the application of genome editing. This may enable a greater range of fruit color options and greater flexibility to cater to consumer preferences around the world.

#### 4.2.6 Nutritional content

Strawberries have a diverse nutritional composition with high levels of biological compounds and phytochemicals (Giampieri et al., 2012; 2013; 2014; Afrin et al., 2016). Strawberries have also been studied for their clinical effects (Afrin et al., 2016). Pigments often add to both nutritive value and antioxidant content (Kapoor et al., 2022). Despite the role of nutritional quality in strawberry popularity, genes underlying nutritional content mechanisms are not widely studied. However, recently, some groups have begun to focus on methods to increase nutritional quality. Integration of wild genotypes into a breeding germplasm has been shown to facilitate improvements in fruit nutritional content (Diamanti et al., 2012; Diamanti et al., 2014). While studies have identified genetic components underlying differences in nutritional quality but have not reported candidate genes or loci (Capocasa et al., 2008; Tulipani et al., 2008), transcriptomic and metabolomic analysis of strawberry development and ripening identified numerous genes associated with the flavonoid pathway, including several associated with ellagitannins and anthocyanins (Baldi et al., 2018).

Application of biotechnology in the improvement of crop nutritional quality has occurred in several other crops and may help to guide future nutritional improvement of strawberry fruit. Overexpression of *AtGalUR* resulted in increased vitamin C content in *Arabidopsis thaliana*, and vitamin C levels in strawberry were found to correlate with expression of the native *AtGalUR* ortholog, indicating the potential to enhance vitamin C content in cultivated strawberry (Agius et al., 2003). Additionally, knockout of *Lycopene Beta Cyclase* (*SlCYCB*) resulted in dark red fruits as result of increased lycopene accumulation (Zsögön et al., 2018). Transgenic insertion of *Narcissus pseudonarcissus Phytoene Synthase* (*NpPSY*) and *Erwinia uredovora Carotene Desaturase* (*EuCRTI*) resulted in β-carotene production in rice (Paine et al., 2005). PSY and Orange (OR) have additionally been identified as key proteins in carotenoid biosynthesis in *Arabidopsis thaliana* (Zhou et al., 2015). Several other carotenoid biosynthesis genes have also been identified in strawberry (Zhu et al., 2015), which may be useful candidates for further investigation of increasing nutritional quality.

#### 4.2.7 Fruit ripening and firmness

Strawberries are non-climacteric, meaning they will continue to redden and soften after harvest, but their flavor will not improve (Azam et al., 2019). In strawberry and other non-climacteric fruits, abscisic acid has demonstrated strong impacts on ripening and softening processes (Li et al., 2011). The popular red “fruit” of strawberry is not a true fruit; it is instead an accessory fruit which is derived from an organ known as the receptacle, a modified stem tip (Hollender et al., 2012; Zhou et al., 2021). The true fruit of a strawberry plant are called achenes, and are the small structures located on the surface of the receptacle (Hollender et al., 2012) which consumers commonly mistake for seeds. Despite this, strawberries are typically used as a model system to study ripening, as plants are small, easy to propagate, have a short vegetative phase, and undergo rapid development and ripening (Perkins-Veazie, 1995; Symons et al., 2012). In this review, ripening is considered the parallel processes of color change and softening. Firmness, while directly associated with the ripening process, differs between genotypes at peak maturity and has a significant impact on postharvest handling and shelf-life.

Numerous genes associated with ripening and softening have been identified and functionalized in both diploid and octoploid strawberry. Downregulation and knockout of *Ripening Inducing Factor* (*FaRIF*) resulted in delayed ripening of both the receptacle and achenes (Martín-Pizarro et al., 2021; Li et al., 2023b). *Sucrose Nonfermenting1-Related Protein* (*FaSnRK2.6*) and *Brap2 Ring ZnF UBP Domain-Containing Protein* (*FaBRIZ*) were found to promote ripening when silenced (Han et al., 2015; Wang et al., 2023a), while downregulation of *Polygalacturonase 1* (*FaPG1*), a β-galactosidase gene (*FaβGAL4*), and a pectate lyase gene (*Fanjjs25*) resulted in increased firmness and reduced postharvest softening (Jiménez-Bermúdez et al., 2002; Garcia-Gago et al., 2009; Quesada et al., 2009; Posé et al., 2013; Paniagua et al., 2016; Paniagua et al., 2020; Paniagua et al., 2022). Knockout of *FaPG1* and *FvSEP3* resulted in significant increase in firmness and reduced postharvest softening, and delayed ripening, respectively (Pi et al., 2021; López-Casado et al., 2023). Several members of the *WRKY* transcription factor (TF) family have also been implicated in strawberry ripening. Overexpression of *FaWRKY71* resulted in increased anthocyanin content and expression of softening-related enzymes (Yue et al., 2022), and transgenic lines for *FvWRKY48*-RNAi displayed significant delays in both fruit development and ripening, as well as increased fruit firmness (Zhang et al., 2022). Lastly, knockout of *FvWRKY50* resulted in delayed anthocyanin accumulation and ripening, though effects on fruit firmness and softening were not reported (Chen et al., 2023). In tomato, knockout of *Pectate Lyase* (*SlPL*) resulted in firmer fruits, and knockout of *Polygalacturonase 2a* (*SlPG2a*) and *β-Galactanase* (*SlTBG4*) resulted in a decrease in pericarp color index (Wang et al., 2019). In peach, virus-induced gene silencing of *Sepallata* (*PrupeSEP1*) resulted in delayed softening of fruits (Li et al., 2017), and in cherry, silencing of *PaMADS7* resulted in inhibited fruit ripening (Qi et al., 2020).

#### 4.2.8 Resistance to common postharvest diseases

Strawberries suffer from numerous postharvest challenges. The thin epidermis of the strawberry receptacle leads to a propensity for mechanical damage, which can occur at all stages of growing, harvesting, and shipping (Gol et al., 2013; Sasaki et al., 2022; Quarshi et al., 2023). Strawberries also frequently undergo rapid softening after being harvested. The combination of these factors further translates into significant susceptibility to pathogens, including *Botrytis cinerea*, *Rhizopus stolonifera*, *Mucor* spp., *Colletotrichum* spp., and *Penicillium* spp. (Vu et al., 2011; Gol et al., 2013; Feliziani and Romanazzi, 2016). Of the numerous diseases known to affect strawberries after harvesting, *Botrytis cinerea*, also known as Botrytis fruit rot (BFR) or Gray Mold, is the primary disease responsible for postharvest loss. *Botrytis cinerea* was once considered the second most important plant fungal pathogen in the world due to its wide host range and ability to cause significant crop damage during both pre- and postharvest (Dean et al., 2012). Because postharvest diseases are major contributors to postharvest loss of strawberry, it is essential to continue improving postharvest disease resistance.

Overexpression of *BRI1-Associated Kinase 1* (*FaBAK1*) and *FaWRKY11* resulted in increased BFR resistance through promotion of defense pathways (Wang et al., 2021b; Li et al., 2023a), and RNAi of *FaWRKY29, FaWRKY64*, and *FaWRKY25* resulted in significant increases in resistance to *Botrytis cinerea* through regulation of other defense-response genes (Wu et al., 2005a; Lee et al., 2023). A total of 247 *WRKY* TFs have been identified in *Fragaria ×ananassa* (Garrido-Gala et al., 2022). Members of the *WRKY* TF family have been characterized for various roles in biotic and abiotic stress response in several crop species (Wu et al., 2005a; Bai et al., 2018; Lee et al., 2023). Thus, further investigation of the roles of *WRKY* TFs in postharvest disease resistance, especially resistance to *Botrytis cinerea*, may be beneficial for the improvement of strawberry postharvest disease resistance. Knockout of *PG1* increased resistance to BFR, possibly due to higher cell wall integrity and reduction of water loss associated with increased firmness (López-Casado et al., 2023). Similarly, RNAi of *β-Glucosidase 3* (*FaBG3*) and *Two-Pore K* ^*+*^ (*FaTPK1*) resulted in increased fruit firmness, delayed ripening, and increased resistance to BFR (Li et al., 2013; Wang et al., 2018), further supporting the impact of fruit firmness on BFR resistance. Multiple volatile compounds have also been tested for their effects on postharvest disease resistance. Methyl anthranilate and γ-decalactone, two major components of strawberry flavor, displayed antipathogenic activity against numerous common strawberry pathogens, including several of postharvest significance (Chambers et al., 2013). Additional exploration of the mechanisms underlying these increases in postharvest disease resistance may reveal targets for genome editing which will enable the improvement of resistance in parallel with other key fruit traits, such as firmness and flavor.

In tomato, knockout of *Mitogen-Activated Protein Kinase* (*SlMAPK3*), *SlMYC2*, and *Autophagy-Related Gene 5* (*SlATG5*) resulted in increased susceptibility to BFR (Zhang et al., 2018; Shu et al., 2020; Li et al., 2023c), whereas knockout of tomato *Phospholipase C2* (*SlPLC2*) increased resistance to BFR (Perk et al., 2023). Virus-induced gene silencing of *RcWAK8* also significantly increased susceptibility to *Botrytis cinerea* in rose (Wang et al., 2023c). Transcriptomic analysis revealed that multiple *FvWAK/WAKL* genes were upregulated during *Botrytis cinerea* infection in strawberry, and which may contribute to BFR resistance (Wang et al., 2023c).

### 4.3 Potential applications of tools developed in strawberry to other rosaceous fruit crops

*Rosaceae* is composed of more than 100 genera and 3,000 species divided into several subfamilies (Potter et al., 2002; 2007; Shulaev et al., 2008), and wide genotypic and physiological variation within *Rosaceae* indicate a need for species-specific transformation protocols for crop improvement (Aldwinckle and Malnoy, 2009). However, if the focus is gene validation, then it would only be necessary to utilize a handful of species as model systems. Among the *Rosaceae* family, the genera *Malus* and *Fragaria* demonstrated the highest transformation efficiencies (Aldwinckle and Malnoy, 2009), indicating their potential value as model systems for reverse genetics in *Rosaceae*. Compared to *Malus*, *Fragaria* has a shorter transformation and regeneration timeline, with production of transgenic lines occurring in as little as 2 months, in addition to a shorter juvenile period (Folta et al., 2006; Aldwinckle and Malnoy, 2009). *Fragaria vesca* has several additional advantages over other plant model systems due to the ability to study mechanisms underlying fleshy fruit development, non-climacteric ripening, and unique metabolites, in addition to having one of the smallest genomes of cultivated plants (Folta and Davis, 2006; Shulaev et al., 2008). For these reasons, tools developed in *Fragaria vesca* may be beneficial to translational studies within Rosaceous species with long periods of juvenility, difficult transformation processes, or other barriers to genomics-assisted improvement. While certain disease resistance and woody plant architecture traits may be difficult to study in a *Fragaria* model (Aldwinckle and Malnoy, 2009), Vilanova et al. (2008) identified sufficient rates of genome synteny between *Fragaria* and *Prunus* to potentially allow for translational studies using marker genes and QTLs developed in strawberry. Others have found high rates of synteny between *Rosa chiloensis* and *Fragaria vesca* (Saint-Oyant et al., 2018), which further supports the use of strawberry as a model system for the development of various genomics and genomics-assisted tools for *Rosaceae*.

With recent increases in reference genome availability for various Rosaceous species came more interest in utilizing family-level approaches to identify loci underlying traits of agronomic interest. One such example of this application was reported by Zurn et al. (2020), in which sweetness-associated genes from *Fragaria* and *Malus* were used to identify a QTL associated with sugar content in blackberry, despite the established lack of blackberry-specific genomic resources. This report highlights the potential applications of marker genes and QTLs developed in widely studied species, such as strawberry, for genomics-assisted improvement of other Rosaceous species with fewer available genomic resources.

## 5 Regulation and commercialization of genome edited strawberry

As with other crops, commercial release of genome edited strawberry will depend on the regulatory frameworks established by individual countries or regions. Starting with the release of the framework 7 CFR Part340^2^ in 1987, genome edited and other genetically plants in the United States were regulated as part of a coordinated framework which includes the U.S Department of Agriculture Animal and Plant Health Inspection Service (USDA-APHIS), the U.S Environmental Protection Agency (EPA), and the U.S Food and Drug Administration (FDA) (United States Department of Agriculture, 2022; United States Food and Drug Administration, 2024). Seven CFR Part 340 underwent revisions in 2020, which included several updates to the existing regulatory system based on 3 decades of research and experience (United States Department of Agriculture, 2022). These updates provide a better breakdown of eligibility for non-regulated status. Application of CRISPR/Cas genome editing strategies can meet multiple of these eligibilities by introducing targeted single base-pair substitutions (7 CFR 340.1(b) (2)) or modifications which are present within the plant’s gene pool (7 CFR 340.1(b) (3)). In addition to the United States, several countries around the world have begun loosening restrictions on genome edited plants (Menz et al., 2020). While the European Union was slower to adopt looser regulations, the European Parliament recently voted to lessen regulatory oversight (Stokstad, 2024), representing a major step forward for global regulation of plant biotechnology.

At this time, there are no commercially available genome edited strawberry varieties. However, other edited species have undergone commercialization. In 2016, non-browning mushrooms were the first CRISPR/Cas9 genome edited organism to pass USDA regulation (Waltz, 2016). Pairwise recently released genome edited *Brassica juncea* with reduced pungency for improved flavor (Brown, 2023). Pairwise has also recently received nine new exemptions from USDA regulation for berry crops, bringing the company’s confirmed exemptions to 19 for berries and 21 in total (Barefoot, 2024). However, these genome edited berries have not been commercially released at the time of this review. These examples and others suggest a bright future for the de-regulation and commercialization of genome edited crops.

## 6 Conclusion and future perspectives

Cultivated strawberries are polyploid, highly heterozygous, and clonally propagated, which makes them difficult to improve through conventional methods. While application of genome editing in diploid woodland strawberry, *Fragaria vesca*, is helpful for validating gene function and identifying new candidate genes underlying traits of interest, the allo-octoploid cultivated strawberry, *Fragaria ×ananassa*, is the species of economic interest. Due to greater genomic complexity and the presence of homoeologous diploid subgenomes which are not closely related to *Fragaria vesca*, gene functions observed in woodland strawberry may not be completely conserved in octoploid strawberry. Thus, validation of gene function in the octoploid background is necessary for further elucidation of pathways underlying traits of interest. In turn, greater understanding of these pathways may then enable precise improvement of strawberry fruit quality and production traits through the application of genomics-assisted breeding techniques or the application of genome editing. Additionally, application of techniques such as base editing and prime editing to make precision nucleotide substitutions, or leveraging HDR to substitute alleles for superior variations, will enable additional opportunities for genomic improvement of strawberry.

While the application of genome editing for crop improvement is still relatively new to *Fragaria ×ananassa* and faces its own significant challenges, several examples of successful genome editing have been reported. These successful reports demonstrate the significant potential of genome editing in strawberry, highlighting the necessity to continue optimizing genome editing methods for improvement of economically important fruit traits. Based on the results of genome editing in *Fragaria vesca*, it may be beneficial to perform editing of orthologs of these genes in octoploid strawberry, to further confirm their roles in the commercially relevant species. Based on the results of transient analyses, some genes associated with desirable fruit traits may also be promising targets for genome editing. In the case of genes which have demonstrated negative effects on desirable traits when knocked out or transiently suppressed, further elucidation of their related pathways may reveal other associated genes which are better targets for application of genome editing and/or may permit fine-tuning of important fruit traits.

## Footnotes

1
https://www.rosaceae.org/organism/Fragaria/x-ananassa?pane=resource-4

2
https://www.ecfr.gov/current/title-7/subtitle-B/chapter-III/part-340
